# Effect of Root Canal Therapy on the Success Rate of Teeth with Complete Roots in Autogenous Tooth Transplantation

**DOI:** 10.1155/2021/6675604

**Published:** 2021-04-14

**Authors:** Xuanyou Cui, Naiyu Cui, Xuehan Li, Xin Du, ShuXin Zhang, Changchun Wu, Dong-Hyuck Kim, Ho-Kyung Lim, Eui-Seok Lee

**Affiliations:** ^1^Department of Oral and Maxillofacial Surgery, Graduate School of Clinical Dentistry, Korea University, Seoul 02841, Republic of Korea; ^2^The Conversationalist Club, School of Stomatology, Shandong First Medical University, Tai'an, Shandong 271016, China

## Abstract

**Background:**

Autogenous tooth transplantation is a reliable method for repairing missing teeth. Although it recently became a recognized and feasible treatment method in dentistry, the long-term efficacy of root canal therapy (RCT) has not been well confirmed. This study is aimed at determining whether RCT has a good effect on the success rate of teeth with complete roots in autogenous tooth transplantation.

**Materials and Methods:**

Data were collected from the Korea University Guro Hospital. Data of patients who underwent autogenous tooth transplantation within 9 years were collected. We selected 29 teeth with complete roots as the research subjects in our study. None of the patients had any systemic diseases. All cases in this study were obtained with patient permission.

**Results:**

According to the tooth vitality test, the autogenous teeth tested negative in dental pulp test and function. Nine of the 29 teeth with autogenous tooth transplantation were treated with RCT and survived throughout the observation period. However, 20 autogenous teeth were not treated with RCT, and eight of them did not survive. All statistical analyses were performed using IBM SPSS 20.0. The null hypothesis was rejected (*p* < 0.05).

**Conclusion:**

For complete root teeth, the success rate after autogenous tooth transplantation after RCT is higher than that of teeth not treated with RCT.

## 1. Introduction

Loss of dentition is considered one of the most common clinical problems for medical staff. Autogenous tooth transplantation refers to the surgical transplantation of nonfunctional teeth to other parts of the same oral cavity such that it can grow and survive in the other alveolar socket and replace the physiological function of the missing tooth. Autogenous tooth transplantation is a treatment method for embedded or heterotopic eruption, early tooth loss, and congenital teeth. It was first recorded in the book *Le Chirurgien Dentiste pub* published by French doctor Pierre Fauchard in 1728 [1–3]. When autogenous tooth transplantation was developed in the early 1950s, the success rate was only approximately 50% due to technical limitations. After a century of development, it has now developed into a safe and predictable technology, and the clinical success rate of autogenous tooth transplantation has reached 75.3–91% [4]. In clinical practice, we often use third molars [5–7], premolars [8, 9], and incisors [10] for autogenous tooth transplantation. As a treatment method for dentition defects, autogenous tooth transplantation replaces the missing teeth with the nonfunctional third molars, retaining the intact periodontal ligament (PDL), possessing physiological mobility and good tissue adaptability and can bear and adjust greater bite force. Compared with implants, autogenous teeth have relatively less occlusal wear and the potential to restore normal periodontal tissue in the receiving tooth area. The treatment cycle is short, the cost is low, and it is easier to accept by most patients [11–13]. Autogenous tooth transplantation usually involves extraction of the affected tooth, followed by removal of the donor tooth. The donor tooth is immediately placed in saline, and the donor tooth is placed in the socket of the missing tooth. Considering surgical complications such as root resorption and ankylosis, autogenous tooth transplantation also has certain limitations [14, 15].

The success of autogenous tooth transplantation is influenced by many prognostic factors, including PDL healing, root development stage, age, multiple suture fixation, and root canal therapy (RCT) history [11, 16, 17]. Current research shows that maintaining a complete and good PDL is vital for successful healing [18]. PDL cells differentiate into dental osteoblasts on the surface of the transplanted root and induce bone regeneration, thereby preventing root resorption [19]. Therefore, reducing the iatrogenic damage of the donor PDL is very important to surgical success. The length of time that the donor teeth are exposed to the body, whether the transplanted tooth root matches the receiving alveolar socket, postoperative fixation, and other factors can affect PDL activity [12]. The principle of sterility should be followed during operation. Many reports have shown that the success rate of a single-rooted teeth transplant is higher than that of multi-rooted teeth [16, 20, 21]. Exposure of the donor tooth outside the mouth for more than 30 min can significantly damage PDL activity and even cause inflammatory root resorption [22]. Inconsistency between the donor tooth and the prepared socket can reduce PDL activity [23]. In recent years, based on cone-beam computed tomography images, a donor tooth model was designed and fabricated using computer-aided design/computer-aided manufacturing, which can avoid unnecessary PDL injury and decrease the donor tooth fitting times [18, 24].

To avoid apical resorption due to crushing infection and inflammation after transplantation, RCT for complete donor teeth can be performed 2 weeks after the operation [11]. The 2-week transition period was chosen to reduce the PDL damage in the initial healing period; if the time is too long, surgical complications of apical resorption may occur [16].

This study retrospectively analyzed the clinical data of 29 teeth admitted to Korea University Guro Hospital for 9 years from 2011 to 2019, providing a reference for follow-up clinical treatment. The stage of root development of the donor tooth is a key factor in the success of autogenous tooth grafting. Autografted teeth with incomplete root formation have the potential for pulp healing to sustain root growth. For teeth with full roots, it is generally believed that root canal treatment should be administered after surgery [6, 14, 25, 26]. Therefore, we chose teeth with complete root formation as the research subject to explore whether RCT had a significant effect on the success rate of teeth with complete roots in autogenous tooth transplantation by analyzing clinical cases and analyze the influencing factors of complete root. The null hypothesis is that RCT has no significant effect on the success rate of teeth with complete roots in autogenous tooth transplantation. The surgical approach and clinical observations are described below.

## 2. Materials and Methods

### 2.1. Cases

This experiment was approved by the Ethical Committee (number 2015GR0706). In this study, 29 cases of autogenous tooth transplantation were performed in the Department of Oral and Maxillofacial Surgery from 2011 to 2019 at Korea University Guro Hospital. A total of 29 teeth were transplanted with autogenous teeth. The longest follow-up period was 9 years. The data of transplanted teeth are shown in Table 1, which shows the total number of teeth with RCT and no RCT.

### 2.2. Case Selection Criteria

All candidates were in good health, followed the postoperative doctor's instructions, and accepted follow-up. The candidates had good oral hygiene. Selecting teeth with fully complete roots as the research object based on radiographic examination is the most important factor. All cases in this study were obtained with patient permission.

### 2.3. Surgical Procedure

A skilled operator performed all transplantation procedures under local anesthesia. In 29 cases, tooth extraction and autotransplantation were performed simultaneously. Before the surgical procedures, clinical and radiographic tests were performed to confirm the condition of the alveolar socket at the receptor, donor site, and depth of impaction. After intraoral disinfection with Betadine, 2% lidocaine HCl capsules containing 1 : 100,000 epinephrine (Yuhan Co., Republic of Korea) were injected around the donor and recipient sites to provide local anesthesia and hemostasis. Recipient site preparation was performed using a low-speed bur with sterile saline irrigation, and in some cases, osteotomy with a chisel was applied. Subsequently, tooth extraction was performed with an extraction forceps as atraumatically as possible to avoid damaging the PDL, including Hertwig's epithelial root sheath (HERS), which remained at the root apex. The extracted tooth was repositioned at the recipient site and fixed for 2 weeks. Different fixing methods were chosen according to the initial stability of the autogenous teeth. For an autogenous tooth with better initial stability, a silk suture was chosen to enhance its stability, while for the one with the worst initial stability, steel wire ligation was chosen to enhance its stability. After fixation, the occlusal relationship was checked to ensure that the transplanted tooth had no early contact or occlusal interference. The use of chlorhexidine gluconate gargle was encouraged.

Two weeks later, the doctor described the risks and benefits of RCT in detail to the patient and decided whether to perform RCT according to the patient's wishes. Root canal preparation was performed using the ProTaper procedure, and the cavity was washed alternately with sodium hypochlorite and EDTA for cleaning and disinfection. Finally, hot vertical pressure filling technology was applied to fill the root canal.

### 2.4. Clinical and Radiographic Examination

Radiographic examination was done before surgery. Based on the classification proposed by Moorrees et al., the developmental stages of the roots of each transplanted tooth during autogenous transplantation were evaluated to ensure that the selected root was fully completed [27]. The roots of the transplanted teeth were evaluated and considered developed when there was partial or no arrest of root formation in teeth with preoperative developmental stages 1–4 (from the beginning of root formation to 3/4 of the expected total length) and apex closure in teeth with preoperative developmental stages 5–7 (full expected total length with varying apex closure).

**Figure 1:**
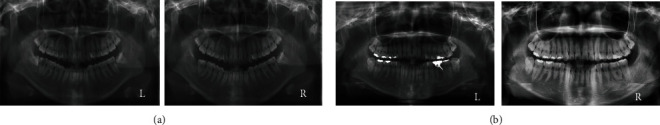
Panoramic tomography images of cases in which the third molars replaced the decayed teeth (L, before transplantation; R, after transplantation). (a) A mandibular third molar replaced a mandibular second molar (L, before surgery; R, after surgery). (b) A mandibular third molar replaced a mandibular first molar (L, before surgery; R, after surgery).

**Figure 2:**
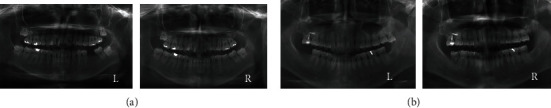
Panoramic tomography images of cases in which the third molars replaced the missing teeth (L, before transplantation; R, after transplantation). (a) A maxillary third molar acted as a mandibular first molar (L, before surgery; R, after surgery). (b) A maxillary third molar acted as a mandibular second molar (L, before surgery; R, after surgery).

**Figure 3:**
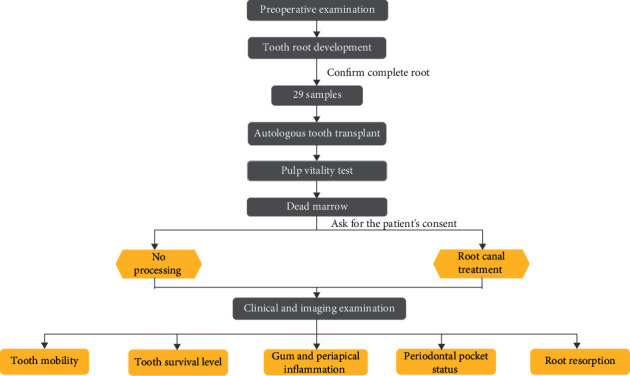
Schematic diagram of the surgical procedure.

At 1 week, 1 month, 3 months, and 6 months after surgery, the same surgeon conducted clinical and radiographic examinations of the transplanted teeth, including tooth mobility, tooth viability, and periodontal pocket status. Periapical radiographs of the transplanted tooth and panoramic radiographs were obtained at each visit. Root resorption, alveolar bone resorption, and periodontal inflammation were assessed (Figures 1–3).

According to the classification proposed by Lindhe et al. [28], tooth mobility was graded as follows: degree #1: tooth mobility within 0.2–1.0 mm in the horizontal direction; degree #2: tooth mobility more than 1.0 mm in the horizontal direction; and degree #3: tooth mobility in the vertical direction.

To conduct the pulp vitality test after surgery, the probe of the pulp vitality tester was coated with a conductive medium and placed on the surface of the tooth, which was wet and dry. The intensity of the released current slowly increased. Once the patient felt numbness, the results were recorded.

For the periodontal pocket test, a periodontal probe was inserted into the periodontal pocket parallel to the main axis of the tooth with a force of 20–30 gf, and a pocket depth of more than 3 mm was considered diseased. Gingival inflammation was scored using the gingival index of Loe and Silness (0.1–1.0, defined as mild; 1.1–2.0, moderate; and 2.1–3.0, severe) [28].

The criteria for success were as follows: (1) no pain after surgery, (2) no progressive root resorption, (3) absence of a morbid periodontal pocket, (4) no significant alveolar bone resorption, and (5) no remaining periapical inflammation. When any of the above occurs, autotransplantation is considered failure despite survival of the transplanted tooth [29].

### 2.5. Statistics

As this study was a qualitative experiment, the chi-square test was applied. The results of the data analysis (Table 1) were statistically analyzed. When *p* was less than 0.05, RCT was considered to have a significant impact on the success rate of autogenous tooth transplantation. All statistical analyses were performed using IBM SPSS 20.0.

## 3. Results and Discussion

The mean postoperative follow-up duration was 36 months. According to the imaging analysis during the follow-up period, the transplanted teeth undergoing RCT achieved PDL healing and bone regeneration. However, some of the transplanted teeth without RCT had pathological phenomena such as abnormal periodontal space and progressive root resorption. Among the 29 transplanted teeth in this study, eight of the 20 teeth that did not receive RCT were removed, while all nine teeth that had been treated were in good condition (Table 1). According to the statistical results, RCT can greatly improve the success rate of autogenous tooth transplantation, and the null hypothesis was rejected (*p* < 0.05).

Root compression injuries may lead to alternative root resorption. It is known that root growth depends on the coordinated activities of HERS, dental pulp, and PDL cells. After transplantation, only the roots with HERS can continue to grow. The sheath is stored around the root apex and damaged easily during the transplantation process, which affects growth of the tooth root [30–32]. Zakershahrak et al. proved that tooth roots with complete HERS can improve the success rate of autogenous tooth transplantation [33]. Based on the above two reasons, in this study, we selected teeth with complete roots to transplant. The successful criteria for the transplantation of this type of transplanted teeth are healing of the PDL and alveolar bone. Previous studies evaluated the clinical effect of PDL healing after autogenous tooth transplantation.

The autogenous teeth tested negative on the dental pulp test and function. After the doctor described the risks and benefits of RCT to the patient in detail, he decided whether to perform RCT according to the patient's wishes.

The time of RCT of our transplanted teeth was 2 weeks after transplantation, because the transplanted teeth had excellent stability after 2 weeks. Chung et al. reported that the incidence of root inflammation absorption in transplanted teeth without RCT was twice that of transplanted teeth with RCT at 2 weeks after transplantation [14]. Herrera et al. studied the transplanted tooth without RCT with external inflammatory root resorption after 3 months [15]. Hence, our experimental result that autogenous tooth transplantation with RCT was successful and the fact that some of those without RCT failed was consistent with previous related studies.

Several factors affect the success of autogenous tooth transplantation. These factors have a decisive effect on the prognosis, including PDL integrity, extra-alveolar time of the transplanted tooth, and RCT [12, 34, 35]. PDL healing is a key factor affecting the long-term prognosis of transplanted teeth. The basic principle is that unfavorable PDL healing affects the long-term prognosis of the tooth and influences the success rate of teeth with complete roots in autogenous tooth transplantation [36]. The healing of periodontal tissue is influenced by PDL activity. Therefore, extraction of the transplanted tooth, preparation of the receiving site, or long-term exposure can easily affect the healing of periodontal tissue. The innermost layer of the PDL can protect the transplanted tooth from tooth root resorption; PDL cells can differentiate into osteoblasts [31, 32]. In addition, the resulting periodontal reaction may have various properties, including gum inflammation, tooth root resorption, and tooth stiffness. However, whether periodontal disease has a harmful effect on autogenous transplantation remains controversial [37, 38]. It is necessary to observe transplanted teeth for a long time, and consistent standards can be used in periodontal healing studies.

Previous studies have shown that the viability of PDL exposed to air declines rapidly after the teeth are outside the mouth for 18 min. Therefore, the time for the donor tooth to be extracted from the alveolar socket to be reimplanted in another alveolar socket should be shorter than 18 min [12, 39]. To reduce the influence of the extraoral time of the donor tooth on replantation success, the transplanted tooth was immediately placed in the recipient site after extraction.

Age is also a possible factor affecting autogenous tooth transplantation outcomes. PDL cells decrease with age; however, PDL has the potential to regenerate, which may affect the normal adaptation of the donor tooth at the recipient site [40]. Altonen et al. found that patients younger than 20 years had a higher transplant success rate than those older than 20 years. From this result, young patients had a good prognosis related to their incomplete formation of tooth roots [41]. The better healing effect of young patients may be related to the increased incidence of dental caries and periodontal inflammation [6, 18]. Tsukiboshi et al. [17] showed that age influences the success rate of autogenous tooth transplantation. Since we currently have fewer cases that cannot be grouped by age, we will continue to collect cases in future clinical studies and strive to discuss the impact of RCT on autogenous tooth transplantation in a larger number of cases by age.

Studies have shown that there are no differences in the survival and success rates of different donor teeth. In addition, sex had no significant effect on the success rate of long-term autogenous tooth transplantation (*p* > 0.05). Therefore, this study did not consider the influence of tooth position and sex on autogenous tooth transplantation in the case selection [25, 42].

The initial stability of the donor tooth affects the postoperative healing mode. Proper initial stability allows moderate functional movement and physiological activity of the transplanted tooth. In addition, it can stimulate and increase PDL cells (e.g., fibroblasts, cementoblasts, and osteoblasts) and bone repair [43, 44]. Initial stability refers to the adaptability of the donor and recipient sites without serious movement. Insufficient initial stability is defined as the horizontal movement of the graft exceeding 2 mm after the graft is moved to the recipient site [45]. Kim et al. believed that initial stability could affect early healing, but it had little effect on long-term healing [18]. In our study, a small number of patients experienced insufficient initial stability after surgery. According to the degree of stability, these cases were fixed for 2 weeks and monitored thereafter. To enhance the adaptability between the transplanted tooth and the recipient site and shorten the operation time, in a future study, cone-beam computed tomography images will be taken before surgery and the size of the recipient socket will be adjusted.

There are currently many ways to fix transplanted teeth, and excellent fixation is greatly conducive to early tooth stability and periodontal tissue healing [46–48]. Sutures can be applied to close the gingival band around the transplanted teeth to prevent the entry of infectants, thereby reducing the incidence of inflammation. Bauss et al. studied the application of sutures to fix donor teeth, and the sutures were removed after 7 days. The success rate of autogenous tooth transplantation can reach 92.9% [49].

## 4. Conclusion

This study showed that the success rate of cases treated with RCT was higher than that of cases without RCT after a follow-up of a few years. Thus, RCT has a significant effect on autogenous tooth transplantation of teeth with complete roots.

## Figures and Tables

**Table 1:** Survival of teeth treated with or without root canal therapy in autogenous tooth transplantation.

	Total number of teeth	Number of teeth removed	Number of teeth that survived
Perform RCT	9	0	9
No RCT	20	8	12
All procedures	29	8	21

All the studied teeth had complete roots.

## Data Availability

The data used to support the findings of this study are included in the article.
